# Actin, RhoA, and Rab11 Participation during Encystment in *Entamoeba invadens*

**DOI:** 10.1155/2013/919345

**Published:** 2013-09-24

**Authors:** M. Herrera-Martínez, V. I. Hernández-Ramírez, A. E. Lagunes-Guillén, B. Chávez-Munguía, P. Talamás-Rohana

**Affiliations:** Departamento de Infectómica y Patogénesis Molecular, Centro de Investigación y de Estudios Avanzados del I.P.N., Avenida Instituto Politécnico Nacional No. 2508, Colonia San Pedro Zacatenco, Delegación Gustavo A. Madero, 07360 México City, DF, Mexico

## Abstract

In the genus *Entamoeba*, actin reorganization is necessary for cyst differentiation; however, its role is still unknown. The aim of this work was to investigate the role of actin and encystation-related proteins during *Entamoeba invadens* encystation. Studied proteins were actin, RhoA, a small GTPase involved through its effectors in the rearrangement of the actin cytoskeleton; Rab11, a protein involved in the transport of encystation vesicles; and enolase, as an encystment vesicles marker. Results showed a high level of polymerized actin accompanied by increased levels of RhoA-GTP during cell rounding and loss of vacuoles. Cytochalasin D, an actin polymerization inhibitor, and Y27632, an inhibitor of RhoA activity, reduced encystment in 80%. These inhibitors also blocked cell rounding, disposal of vacuoles, and the proper formation of the cysts wall. At later times, F-actin and Rab11 colocalized with enolase, suggesting that Rab11 could participate in the transport of the cyst wall components through the F-actin cytoskeleton. These results suggest that actin cytoskeleton rearrangement is playing a decisive role in determining cell morphology changes and helping with the transport of cell wall components to the cell surface during encystment of *E. invadens*.

## 1. Introduction

*Entamoeba invadens* causes reptilian amoebiasis resulting almost in 100% mortality. This parasite is similar morphologically [1] and genetically to *Entamoeba histolytica *[2]; the causative agent of the human invasive amoebiasis. *E. invadens* encystment is easily induced *in vitro* [3–5] and for this reason it has been used as model to study encystation for the genus *Entamoeba*.

An initial event on *E. invadens* encystation process involves the formation of large aggregates of trophozoites, which may be required to maintain physical contact or to reach critical levels of autocrine metabolites secreted by trophozoites [6]. When these aggregates are formed, the differentiation of trophozoites into cysts initiates through intracellular rearrangements that lead to cell rounding; also cellular compaction occurs, most probably through the loss of vacuoles and the synthesis and deposition of the cyst wall components [1]. The reorganization of the actin cytoskeleton appears to be essential for cyst differentiation in this parasite [7]. Actin transition between monomeric (G-actin) and filamentous (F-actin) states plays a decisive role in many cellular functions [8]. In the case of *Entamoeba*, a large number of actin-binding proteins that regulate actin assembly by controlling nucleation of monomers, elongation and polymerization/depolymerization of the filaments, and cross-linking of the actin network have been described [9]. Small GTPases, the members of the Rho family, are also involved through their effectors in the control of the cytoskeleton function. It has been suggested that Rho1, through Rho-associated protein kinase 2 (ROCK-2), activates actin microfilament rearrangements induced by fibronectin signaling [10], and most recently, it was found that Rho1 regulates actin polymerization through diaphanous-related Formin1 [11]. 

In eukaryotic cells, Rab proteins are recruited and activated in the donor membrane; they regulate the formation, transport, joining, and fusion of vesicles. The encystation of *Entamoeba invadens* involves synthesis of proteins belonging to encystment vesicles, including enolase [12], and the transportation of these vesicles towards the cell surface to form the cyst wall. In *Giardia lamblia *[13], Rab11 has been shown to facilitate the transport of the cell wall protein 1 (CWP1) through its strong connection with actin cytoskeletal. While Rab11 has been suggested to participate in the *Entamoeba* encystment process [14], to date, there are no reports of its participation during this process.

Based on the above information, the aim of this study was to analyze whether F-actin, RhoA, and Rab11 participate in a coordinated manner during *E. invadens* encystation process. We use total population of asynchronous cultures at trophozoite state (T) and at 12, 24, 48, 72, and 96 h after induction of encystment, and molecules of interest were followed by confocal microscopy, western blot analysis, and G-LISA RhoA activation assays. Also, inhibitors of actin polymerization, such as Cytochalasin D, and of RhoA activity, such as Y27632, were used to verify the participation of actin and actin rearrangements mechanisms during encystation. Results showed that morphological changes, loss of vacuoles, and transport of encystment vesicles are all processes related to the actin cytoskeleton function.

## 2. Materials and Methods

### 2.1. Antibodies

Primary antibodies used in this study were antiactin (MAB 1501 clone C4, Millipore) [10], anti-Rab11 (C-19 sc-6565, Santa Cruz Biotechnology), and antienolase (A-5 sc-271384, Santa Cruz Biotechnology). For western blot analysis, secondary antibody goat anti-mouse conjugated to HRP (31430, Thermo Scientific) was used. For confocal analysis, the secondary antibodies and fluorescent probes used were Pacific Blue goat anti-mouse IgG (H1L) (Invitrogen, P31582), FITC-conjugated mouse anti-goat IgG (31510, Pierce), and rhodamine phalloidin (Invitrogen; R415).

### 2.2. Parasite Cultures and *In Vitro* Encystation

*E. invadens* trophozoites (IP-1 strain) were axenically grown at 26°C in complete TYI-S-33 medium with 10% bovine serum [15]. To induce encystment, trophozoites harvested in the logarithmic phase of growth (5 × 10^5^/mL) were transferred to LG encystation medium (TYI without glucose) diluted to 47% with 5% bovine serum [16]. Cultures were incubated at 26°C, and cells were counted to determine trophozoites, round precysts (20–40 *μ*m), and cysts (10–20 *μ*m) at 12, 24, 48, 72, and 96 h after induction. To confirm cysts formation, the pellet was resuspended, in 1 mL of 0.2% w/v Triton X-100 for 10 min, and the number of detergent-resistant cysts was determined using a haemocytometer.

### 2.3. Fluorescence Microscopy

Samples of the encystment kinetics were fixed with 4% paraformaldehyde for 1 h at room temperature (RT) and were permeabilized with 0.2% Triton X-100 in PBS. Samples of 48, 72, and 96 h were given 3 freezing/thawing cycles [17]. Samples were blocked with 10% BSF/PBS for 1 h and incubated overnight with anti-actin (1 : 1,000), anti-enolase (1 : 50), and anti-Rab11 (1 : 25) at 4°C. Cells were washed with PBS; FITC-labeled mouse anti-goat IgG and Pacific Blue goat anti-mouse IgG were used as secondary antibodies, respectively, (1 : 100 dilution), with each incubation step for 1 h at 37°C. Samples were incubated with rhodamine phalloidin (1 : 50) for 1 h at 37°C and DAPI (1 : 300) (stock 1 mg/mL, D-1306, Molecular Probes, Inc.) for 30 min at 37°C. Samples were mounted with Vecta-Shield medium (Vector Laboratories) and observed in a Carl Zeiss LSM 700 confocal microscope.

### 2.4. Electrophoresis and Western Blot Analysis

Total samples were washed with PBS and resuspended with Tris-HCl 0.05 M/NaCl 0.15 M, pH 7.2 containing 3 mM N-ethylmaleimide (NEM), iodoacetamide (IA), and tosyl-lysilchloromethyl-ketone (TLCK): 1 mM PMSF, 2.5% Triton X-100. The cell suspension was sonicated, at 4°C by 10 min with 5 s of sonication, and 5 s of rest, in an Ultrasonic Cell Crusher Sonic-150 W. Total extract was centrifuged at 7,500 ×g for 20 min at 4°C. Soluble fraction (Sf) was separated from the pellet (If). Protein concentration from Sf and If was determined by Bradford's method with a DC Protein Assay (Bio-Rad 500-0116). Equivalent amounts of protein from both fractions were run in 10% SDS-PAGE under reducing conditions. Proteins were then transferred onto nitrocellulose membranes (Bio-Rad, Hercules, CA), blocked with 10% nonfat dry milk in PBS for 1 h at RT, washed, and incubated overnight at 4°C with an anti-actin antibody (1 : 10,000). Membranes were washed with PBS, and then incubated with goat anti-mouse IgG conjugated to HRP (1 : 1,000) for 2 h at RT. After washing with PBS, antibody-reactive actin was detected by chemiluminescence using the substrate Signal (ECL, Bio-Rad) according to the manufacturer's instructions. Bands were analyzed by the Gene Snap program from SynGene.

### 2.5. Transmission Electron Microscopy

Samples were fixed in 2.5% (v/v) glutaraldehyde in 0.1 M sodium cacodylate buffer pH 7.2 for 60 min. They were postfixed for 60 min with 1% (w/v) osmium tetroxide in the same buffer. After dehydration in increasing concentrations of ethanol and propylene oxide, samples were embedded in Polybed epoxy resins and polymerized at 60°C for 24 h. Thin sections (i.e., 60 nm) were contrasted with uranyl acetate and lead citrate before being examined in a Philips Morgani 268 D (FEI Company, Eindhoven, The Netherlands) electron microscope.

### 2.6. Measurement of RhoA Activity

RhoA activity was determined by using a luminescence based G-LISA RhoA activation assay kit (Kit no. BK121, Cytoskeleton, Inc., Denver, CO) according to the manufacturer's instructions. This assay employs a Rho-GTP binding protein coating the wells of a 96-well plate. Active GTP-bound Rho, in cell lysates, binds to the wells while inactive GDP-bound Rho is removed through the washing steps. Then the bound active RhoA is detected by incubation with a specific anti-RhoA antibody followed by a HRP-conjugated secondary antibody and a detection reagent, after which the luminescence is read on a microplate luminescence reader. Samples were prepared as described. Proteins were obtained by incubating cells with the provided cell lysis buffer with protease inhibitors, and immediately they were clarified by centrifugation at 18,000 ×g at 4°C for 2 min. Protein concentration was determined according to the manufacturer's protocol, and cell extracts were equalized to a protein concentration of 1.0 mg/mL for the assay. Samples were then added to the wells of the Rho G-LISA plate coated with Rho-GTP binding protein. The plate was placed on a cold microplate shaker set at 400 rpm at 4°C for 30 min. The plate was washed three times with Wash Buffer at RT. Then, anti-RhoA primary antibody (1 : 250) was added to each well and the plate was placed on the shaker for 45 min. After three washes, secondary antibody was incubated by 45 min and the HRP detection reagent (provided with the enhanced chemiluminescence kit) was added to the wells, and the luminescence signal was detected by a microplate luminescence reader (Fluoroskan Ascent FL Thermo). A calibration curve with a positive control was done, and results are shown as *ρ*g of RhoA-GTP/*μ*g protein extract.

### 2.7. Effect of Cytochalasin D and Y27632 on *Entamoeba invadens* Encystation

To determine the effect of drugs treatment on trophozoites viability, cells (4 × 10^4^ cell/mL) were grown in complete TYI-S-33 medium with 10% bovine serum at 26°C, in the presence of DMSO (vehicle), cytochalasin D (CD) (1 *μ*M) or Y27632 (1 *μ*M) for 72 h. Trophozoites' viability was determined by trypan-blue dye exclusion. To determine the effect of CD and Y27632 in the efficiency of encystment, axenized trophozoites (5 × 10^5^ cell/mL) harvested during logarithmic growth were transferred to LG encystation medium diluted at 47% [16] and were incubated in the presence of CD (1 *μ*M) or Y27632 (1 *μ*M) for 96 h. The percentage of detergent-resistant cysts was determined by incubation in 0.2% w/v Triton X-100 for 10 min and counted by trypan-blue dye exclusion. Samples of drugs treated cells were taken at 12 h for confocal microscopy analysis and 96 h for electron microscopy analysis.

### 2.8. Statistical Analysis

Data are representative of three independent experiments, each one in duplicate. The significant difference of experimental compared with control samples was analyzed by paired Student's *t*-test (*P < 0.05, **P < 0.001). All statistical analysis was carried out using the statistical program Sigma Plot version 11.0.

## 3. Results

### 3.1. High Identity of *Ei*actin, *Ei*Rab11, *Ei*enolase, and *Ei*RhoA with Homologous Human Molecules

**Figure 1:**
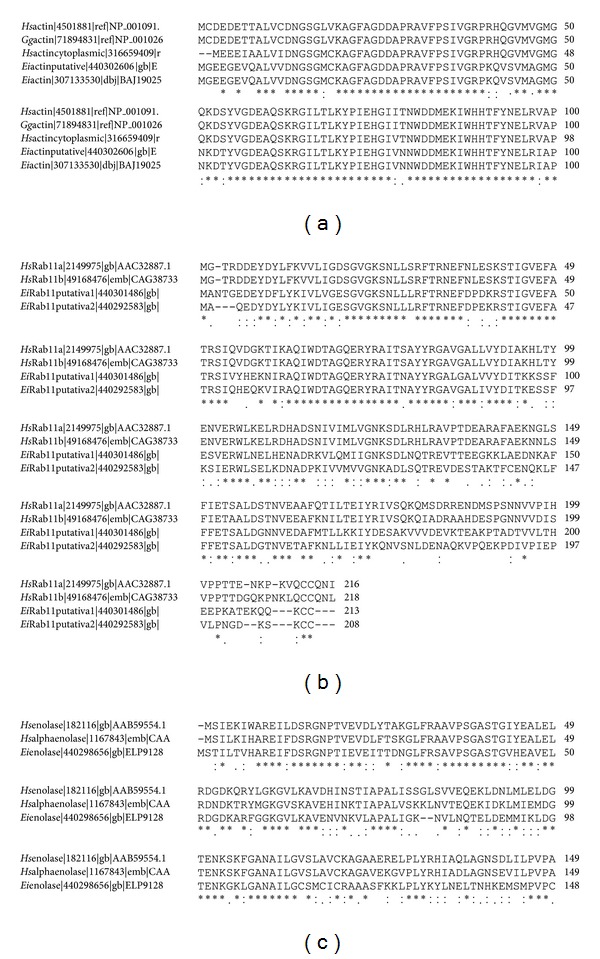
Bioinformatic analysis of sequence homology between *Ei*actin, *Ei*Rab11, and *Ei*enolase with their human counterparts molecules. (a) High identity of N-terminal between *Ei*actin, *Hs*actin, and *Gg*actin is shown. (b) *Ei*Rab11 presented an identity of 54–57% with *Hs*Rab11A on their complete sequence. (c) *Ei*enolase presented high identity of its N-terminal sequence with human enolase residues. Identical residues are shown in “∗”, conserved residues in “:”, and semiconserved residues in “.”.

As a first approach for this study, bioinformatic analysis of the molecules of *Entamoeba invadens* and *Homo sapiens* was done to ensure that heterologous antibodies could be used to track the proteins of interest. Clustal 2.1 multiple sequence alignment program was used; identical residues are shown in “∗”, conserved residues in “:”, and semiconserved residues in “.” (Figure 1). *Ei*actin presented 85–88% identity with *Hs*actin and *Gg*actin (*Gallus gallus*) based on their complete sequences. High identity of N-terminal between all molecules is shown in Figure 1(a). *Ei*Rab11 presented a 54–57% identity with *Hs*Rab11A based on their complete sequences. Identity of complete sequences is shown (Figure 1(b)). *Ei*enolase presented 64% identity with *Hs*alphaenolase on the basis of the complete sequence. High identity of N-terminal between all molecules is shown (Figure 1(c)). Blast analysis (PubMed) of RhoA of human origin was run to find similar sequences in *E. invadens*. First 10 sequences were taken to perform an alignment of them. *Ei*Rho presented 47–51% identity with *Hs*RhoA. Based on the above results, it was possible to predict a substantial cross-reactivity between anti-human proteins antibodies and homologous isoforms in *E. invadens*.

### 3.2. Polymerized Actin Levels Vary along the Encystation Process

**Figure 2:**
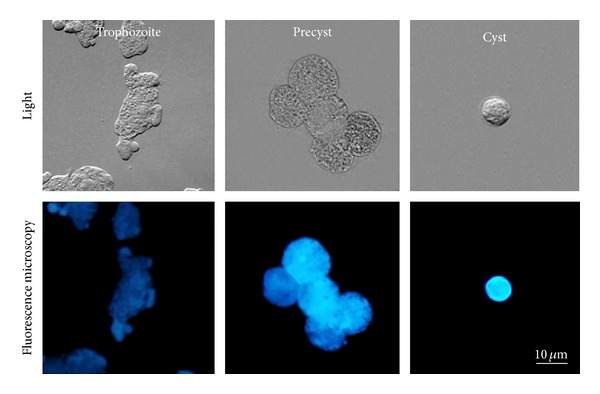
Calcofluor m2r staining of trophozoites, precysts, and cysts. Micrographs show phase contrast and fluorescence images of nonpermeabilized amoebae, stained with calcofluor m2r. Inside precysts, there are fluorescent structures with vesicular appearance, a full staining of the outer cyst wall in a cyst.

In *E. invadens*, trophozoite-cyst transformation is a critical process in which the formation of the cyst wall, composed mainly of chitin microfibrils, is carried out [18].  Figure 2 shows the specific reaction of a fluorescent dye, calcofluor white m2r, with chitin molecules present in the cyst wall.

**Figure 3:**
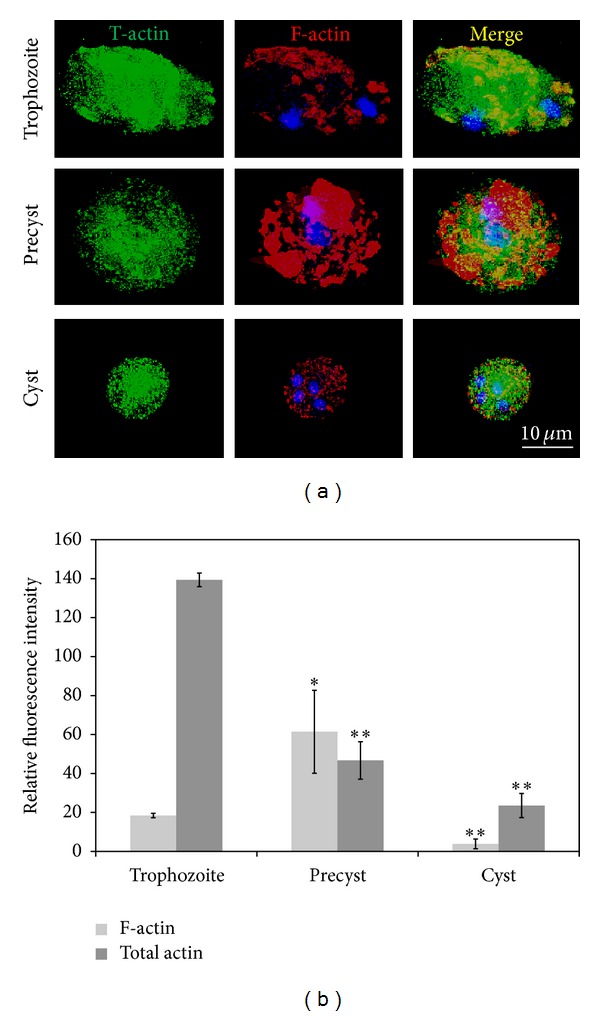
Polymerized actin levels vary along the encystation process. (a) Micrographs showing total (anti-actin antibody 1 : 10,000 followed by a secondary FITC-conjugated goat anti-mouse IgG antibody, 1 : 1,000), and filamentous actin (Rhodamine phalloidin, 1 : 50) in the three developmental stages during encystment. (b) Graph representing the intensity of T-actin and F-actin. T-actin decreases in precyst and cysts, but F-actin increased in precysts. Results are the average of three independent experiments done in duplicate (*P < 0.05, **P < 0.001).

Actin cytoskeleton reorganization is essential for *E. invadens* encystment [7]; however, the extent of its participation has not been determined. In this work, polymerized actin structuration and quantification were studied by fluorescence microscopy in trophozoites, precysts, and cysts. Cells were stained with rhodamine-phalloidin; phalloidin is a mycotoxin derived from the fungus *Amanita phalloides*, which specifically binds to filamentous actin (F-actin). In order to detect depolymerized actin (G-actin), an anti-actin antibody was used in combination with a secondary FITC-conjugated anti-IgG antibody. Total actin decreased during encystment (Figure 3(a)); however, the amount of polymerized actin showed an increase in the precyst state comparing with trophozoites and cysts (Figure 3(b)). This indicates that the actin cytoskeleton polymerization state (polymerized/depolymerized actin ratio) changes during encystment.

To follow changes in actin polymerization during stages conversion, trophozoite to precyst to cyst, trophozoites were induced to encyst and samples were analyzed at 12, 24, 48, 72, and 96 h after induction of encystment.

### 3.3. Increase, in Polymerized Actin Level, in the Early Stages of Encystment

**Figure 4:**
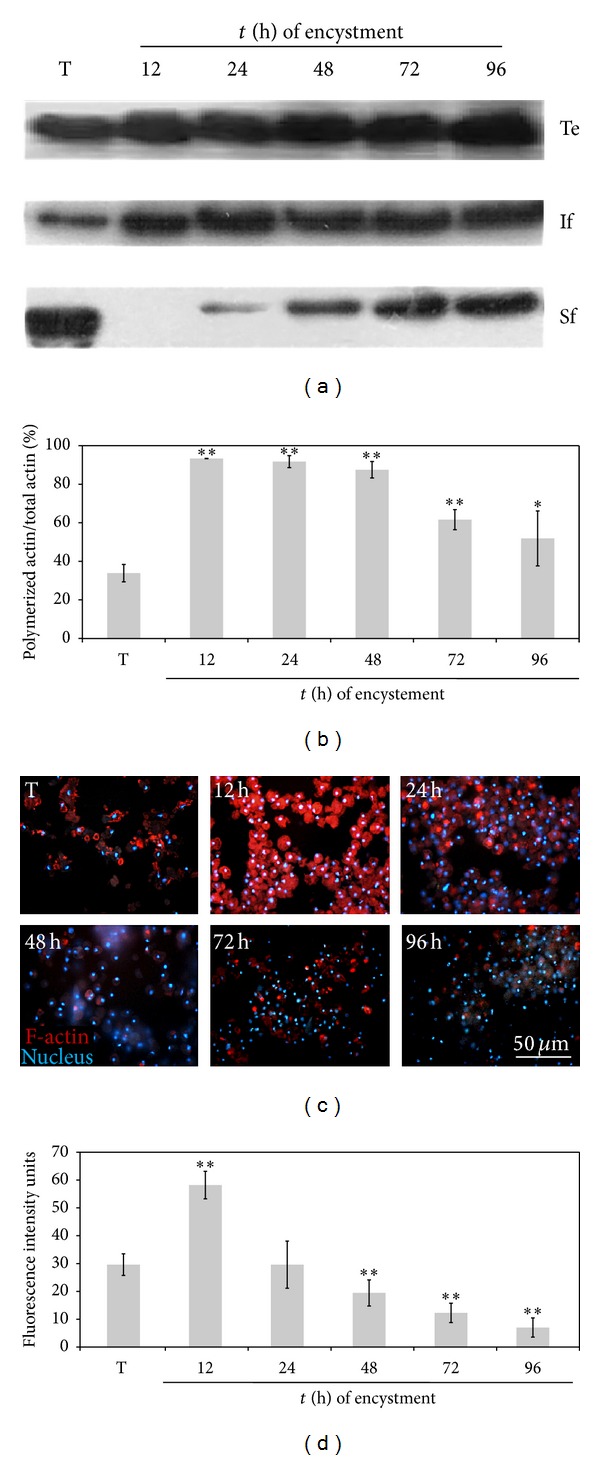
Increase in polymerized actin level, in the early stages of encystment. ((a), (b)) Western blot and densitometric analysis showing an increase in polymerized actin in comparison with total actin [If/(Sf + If)∗100] during encystment and ((c), (d)) an increase of F-actin (red) by fluorescence microscopy at early times after induction of encystment (12 h). Results are the average of three independent experiments done in duplicate (*P < 0.05, **P < 0.001).

There have been controversial reports regarding the amount of actin in trophozoites and cysts; while Manning-Cela et al. (1994) observed a decrease in cysts [19], Makioka et al. (2011) found similar amounts in both phases of the cell cycle of the parasite [20]. As a first step to explain these inconsistencies, actin amount in total extracts from asynchronous cultures that were induced to encystment (T, 12, 24, 48, 72, and 96 h) was analyzed by western blot. Results in Figure 4(a) show that the amount of actin present in total extracts remained the same throughout the encystation process. However, after subcellular fractionation, the amount of actin in the soluble (depolymerized actin) and insoluble (polymerized actin) fractions showed a modification in the polymerized/total actin ratio during encystment (Figure 4(b)). Thus, the ratio of polymerized/total actin was higher during encystment compared to that of trophozoites; the highest level was found at 12 h, time after which this ratio decreases progressively with the time of encystment (Figure 4(b)).

To confirm the state of actin polymerization, cells stained with rhodamine phalloidin were analyzed by fluorescence microscopy. The intensity of staining increased at 12 and 24 h but gradually decreased from 48 to 96 h (Figures 4(c) and 4(d)).

In both cases (Wb and light microscopy), an increase in F-actin was observed, indicating an important actin polymerization dynamic, especially at early times after induction of encystment.

### 3.4. Possible Involvement of Polymerized Actin in the Transportation and Disposal of Vacuoles

**Figure 5:**
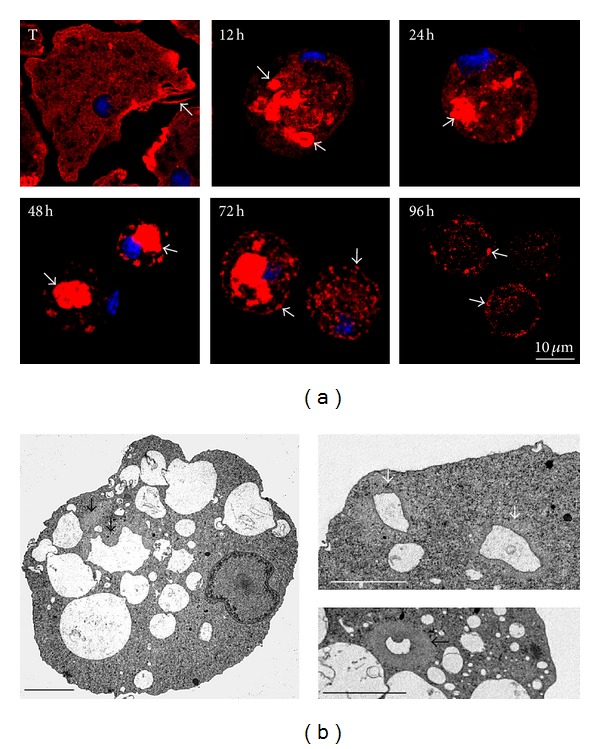
Physical association of actin with intracytoplasmic vacuoles. (a) F-actin is present in filopodia and adhesion plaques in the trophozoite; at 12 h, F-actin is surrounding structures with vacuolar appearance (arrows); at 24 and 48 h, F-actin was concentrated in a region of the amoeba (arrows); at 72 h, F-actin is located in structures with vesicular appearance (arrows); at 96 h, F-actin localized in the periphery of the cyst (arrows). F-actin was detected by Rhodamine-phalloidin (1 : 50) by confocal microscopy. Images are representative of two independent experiments. (b) Ultrastructure of the encystment process. The elimination of vacuoles, surrounded by a cytoplasm rich in thin filaments (arrows), characteristic appearance of high actin content, was observed at early times after induction of encystment (12 h). Samples were processed as described in Section 2.

To analyze the type and location of structures formed by F-actin during encystment, samples were analyzed by confocal microscopy. Trophozoites showed actin filaments abundantly in the periphery of the cell, in filopodia, in adhesion plaques, and in phagocytic structures (Figure 5(a), T). At 12 h after induction of encystment, F-actin appeared surrounding vacuoles (arrows), whereas at 24 h the number of these structures was reduced, at 48 h mostly a single but larger cluster of actin was observed in the center of the amoebas. At 72 h smaller structures of F-actin with vesicular appearance were seen, and at 96 h scarce dots of F-actin were found decorating the periphery of the cysts (Figure 5(a), 12–96 h).

Samples were further studied by transmission electron microscopy. At early times, a noticeable change in morphology was observed, from pleomorphic to round forms (data not shown), together with the elimination of vacuoles (Figure 5(b)), generating compact and rounded amoebas as has been reported [21]. At early times, amoebas showed vacuoles surrounded by cytoplasm, rich in extremely thin filaments (arrows in Figure 5(b)), characteristic appearance, at the transmission electron microscope, of high actin content material [22]. These observations suggest that, early in the process, actin could be involved in the transportation and elimination of vacuoles in the amoebae cytoplasm, which may be consistent with a lesser amount of vacuoles and smaller size of the cell as occurs in the cyst.

### 3.5. Increase in RhoA-GTP Levels at Early Times of Encystment

Despite the lack of reports about the participation of activated RhoA during the encystment in *E. invadens*, it is known that in *E. histolytica*, Rho is essential for actin restructuring during the interaction of the amoeba with fibronectin [10]. The importance of the actin cytoskeleton in the encystment of *E. invadens* led us to determine the levels of the active state of this GTPase.

**Figure 6:**
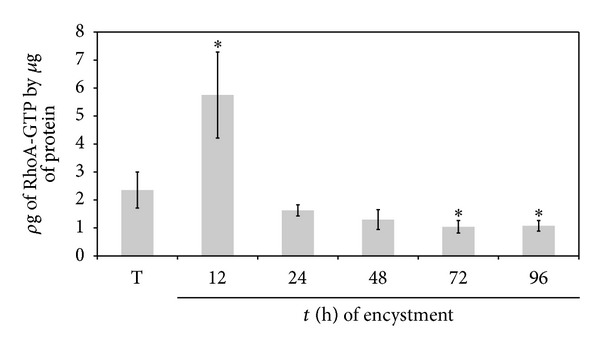
Increase in RhoA-GTP levels at early times of encystment. RhoA-GTP increased at early times but decreased later on during encystment. RhoA-GTP levels were determined by G-LISA RhoA activation assay kit (Kit no. BK121, Cytoskeleton, Inc., Denver, CO). Results are the average of three independent experiments done in duplicate (*P < 0.05).

G-LISA RhoA activation assays showed that, in trophozoites, RhoA-GTP levels were 2.35 ± 0.64 *ρ*g, with a significant increase at 12 h to 5.75 ± 1.54 *ρ*g; at 24 and 48 h RhoA-GTP levels were slightly below levels found in trophozoites (1.63 ± 0.20 and 1.30 ± 0.35 *ρ*g, resp.), but at 72 h (1.04 ± 0.22 *ρ*g) and 96 h (1.07 ± 0.19 *ρ*g) these levels decreased further (Figure 6). The activation of RhoA may indicate the need of the amoeba to regulate the actin cytoskeleton rearrangement during encystment, especially at early times of the process.

### 3.6. Actin Cytoskeleton Participates in the Morphological Change, Transportation and Disposal of Vacuoles, and Formation of the Cyst Wall

**Figure 7:**
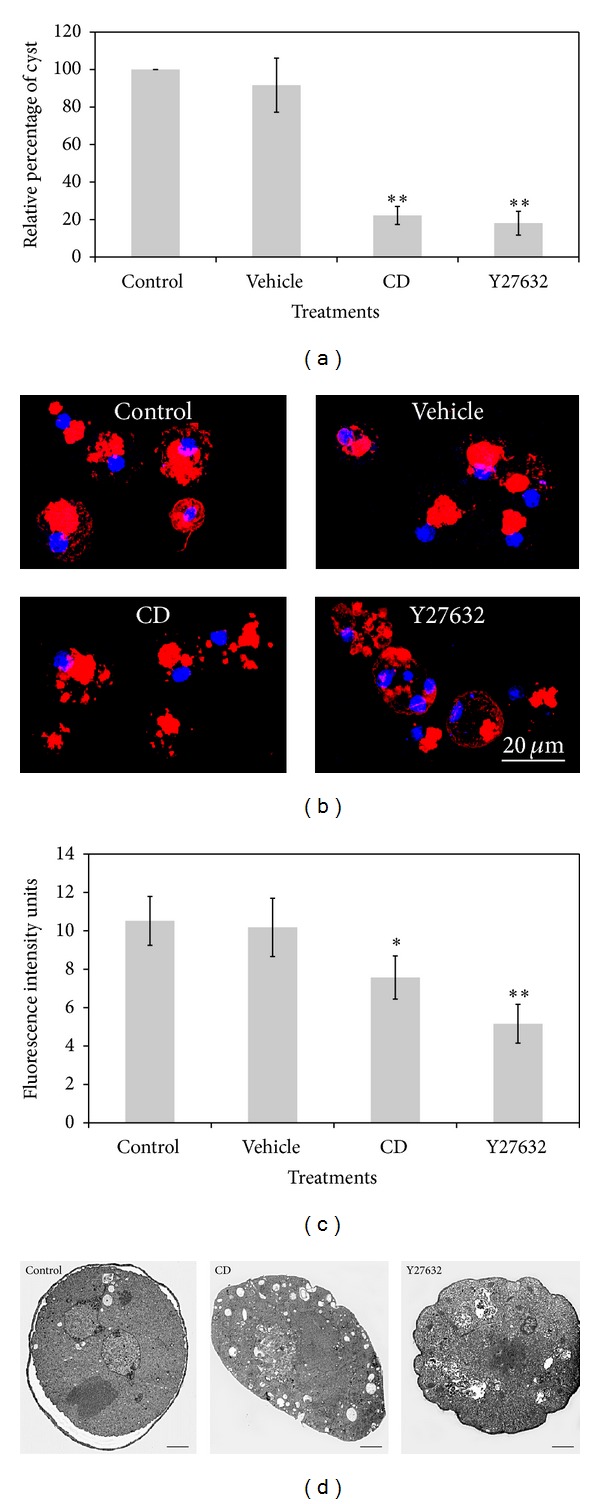
Disruption of the actin cytoskeleton with CD and Y27632 blocked the rearrangement of the actin cytoskeleton strongly affecting the cyst formation. (a) Percentage of inhibition of encystment by CD (1 *μ*M) and Y27632 (1 *μ*M) treatment during 96 h. ((b), (c)) Decrease in the intensity of F-actin fluorescence as a consequence of CD and Y27632 treatment. (d) Cysts ultrastructure at 96 h produced in the absence (control) or the presence of Cytochalasin D (CD) and ROCK-2 inhibitor (Y27632). Bar = 2 *μ*m.

To confirm the participation of the actin cytoskeleton and its rearrangement during *E. invadens* encystment, Cytochalasin D (CD), an actin filaments polymerization and elongation inhibitor [23], and Y27632, a drug capable of selectively inhibiting RhoA kinase (ROCK-2), thereby suppressing induced RhoA activity [24], were used to analyze encystment efficiency (Figure 7(a)), actin polymerization (Figures 7(b) and 7(c)), and cyst wall formation (Figure 7(d)). Drugs used at 1 *μ*M concentration did not have any effect on trophozoites viability after 72 h of treatment (data not shown). However, they strongly inhibited the formation of cysts (Figure 7(a)). CD showed an inhibition of 78.78% while Y27632 an inhibition of 82.94% on cysts formation. These results confirm the importance of actin cytoskeleton rearrangements and the activity of RhoA and RhoA effectors during encystment.

To visualize the actin polymerization state during encystment in the presence of CD or Y27632, the structuration type and amount of F-actin were analyzed at 12 h after encystment induction because this was the time point at which cells presented the highest actin polymerization level. The control condition showed rounded amoebae, round vacuole-like structures formed by F-actin forming large clusters. Amoebae treated with vehicle (DMSO) showed the same structuring pattern as control cells. Treatment with CD formed large clusters of F-actin but also small structures dispersed in the round amoeba while amoebae treated with Y27632 presented irregular morphology and structures of F-actin outlining the cytoplasmic membrane without forming the characteristic clusters of untreated amoebae (Figure 7(b)). F-actin level, measured by rhodamine intensity, showed a slight decrease in amoebae treated with CD and a more pronounced reduction in amoebae treated with Y27632 (Figure 7(c)).

Encystation process involves the formation of large aggregates of trophozoites that lead to cell rounding. Later, vacuoles fuse with each other and then they are transported towards the cell membrane and removed from the cell. Subsequently, synthesis and transport of encystment vesicles happen to carry out the formation of the cyst wall. At the end of the process, cysts with well-formed cyst wall and cytoplasm containing the chromatoid bodies can be observed [21]. This was the case for nontreated cells (Figure 7(d), control), whereas in the presence of CD, amoebae induced to encyst showed the formation of large aggregates of trophozoites; however, these CD treated cells were not able to round up, and their number of vacuoles was higher compared with that of amoebae without drugs or treated with DMSO (data not shown); there was no formation of the cyst wall either (Figure 7(d), CD). In the presence of Y27632, amoebae formed clusters and rounded up (data not shown); they also reduced their number of vacuoles and were able to form the cyst wall, as occurred with nontreated amoebae; however, the cytoplasm showed a thick fibrous appearance (Figure 7(d), Y27632).

These results show that disruption of the actin cytoskeleton with CD blocked the structuration of the actin cytoskeleton, strongly affecting the cyst formation, whereas with Y27632, some events of the encystment process such as the elimination of vacuoles and the proper formation of the cyst wall were affected. These results confirm that the encystation process is highly dependent on different actin cytoskeleton functions through different time points during encystment.

### 3.7. Rab11 Localizes with ENO during Encystment

**Figure 8:**
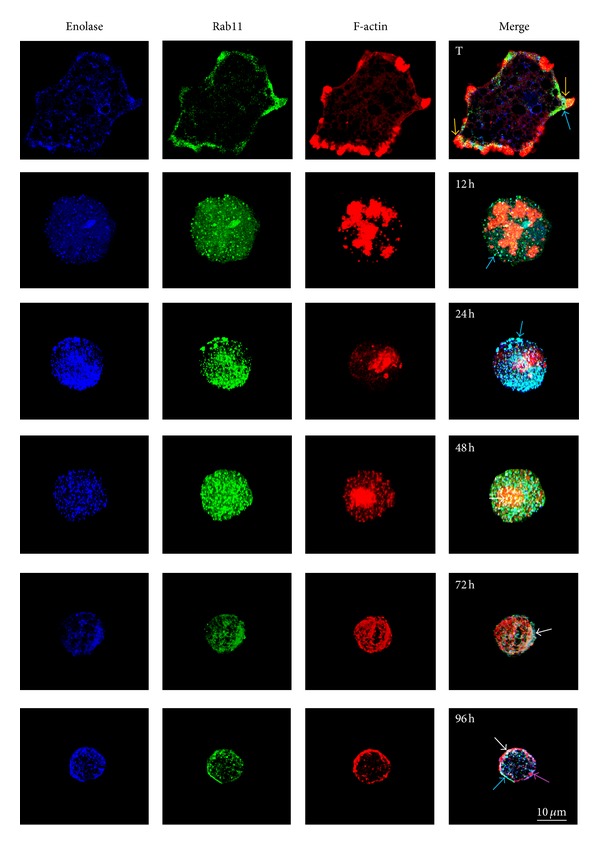
Rab11 colocalizes with ENO during encystment. Trophozoites were induced to encyst by incubation in LG medium diluted to 47% containing 5% ABS. At 12, 24, 48, 72, and 96 h, aliquots were taken, fixed with 4% paraformaldehyde and blocked with 10% FBS, before staining to analyze the localization of enolase with anti-enolase mAb (1 : 50) (A-5, sc-271384 Santa Cruz Biotechnology), Rab 11 with anti-Rab-11 mAb (1 : 25) (C-19, sc-6565 Santa Cruz Biotechnology), and F-actin with Rhodamine phalloidin (1 : 50) (R415, Invitrogen). Cells were viewed with a Carl Zeiss LSM 700 microscope. Micrographs correspond to 3D optical images. Co-localization, of molecules of interest, is shown by arrows.

Rab11 is a protein that participates in recycling during endosomes traffic; besides it participates in the transport of internalized receptors, from the Golgi to the plasma membrane. In *Entamoeba* a putative Golgi apparatus has been described, suggesting that it behaves similarly, with respect to vesicular transport, to Golgi apparatus present in higher eukaryotic cells [25–27]. Results obtained from the comparative genomic analysis of human Rab11 and *Ei*Rab11 (Figure 1) suggest a highly conserved vesicular trafficking machinery. During encystation of *Giardia lamblia*, Rab11 is involved in the transport of CWP1 to the periphery of the cyst through the actin cytoskeleton [13]. Therefore, it is possible to conclude that during encystment of *E. invadens*, Rab11 could also participate in the transport of proteins forming the cyst wall in this parasite. Consequently, the subcellular localizations of enolase (ENO), an enzyme found in encystment vesicles [12], F-actin, and Rab11 were determined during encystment (Figure 8) by confocal microscopy.

In trophozoites (0 h), ENO was dispersed in the cytosol, with Rab11 decorating the plasma membrane and F-actin structuring adhesion plaques at the periphery of the amoeba. At 12 h, ENO and Rab11 were relocalized in structures with vesicular appearance; in many of them, both proteins colocalized (co-localization coefficient 0.66 ± 0.054). At 24 h, the level of expression of ENO and Rab11 increased, and both were located in the same vesicles (arrows) (0.78 ± 0.008). However, F-actin was concentrated in a large body that also colocalized with ENO, although with a relatively low co-localization coefficient (0.173 ± 0.070). At 48 h, F-actin was organized into numerous vesicles that colocalized with ENO (0.099 ± 0.010) and Rab11 (0.119 ± 0.016). At 72 h, F-actin organized into several vesicles that colocalized with the ENO (0.099 ± 0.010 and 0.127 ± 0.033, resp.) and Rab11 (0.119 ± 0.016 and 0.157 ± 0.037). At 72 h, the three proteins were found decorating part of the cyst surface and parts of the cytosol, colocalizing ENO and Rab11 (0.192 ± 0.051), ENO and F-actin (0.127 ± 0.033), and Rab11 and F-actin (0.157 ± 0.037). At 96 h, ENO and Rab11 were decorating the wall of the cyst (0.194 ± 0.146) (Table 1).

From these results, we hypothesize that Rab11 participates in the transport of ENO to the cell wall through F-actin. In this process, F-actin/G-actin transition was particularly dynamic, observing co-localization values between ENO and F-actin up and down.

### 3.8. Performance of RhoA-GTP and Actin during the Transitional Stages throughout Encystment Kinetic

**Figure 9:**
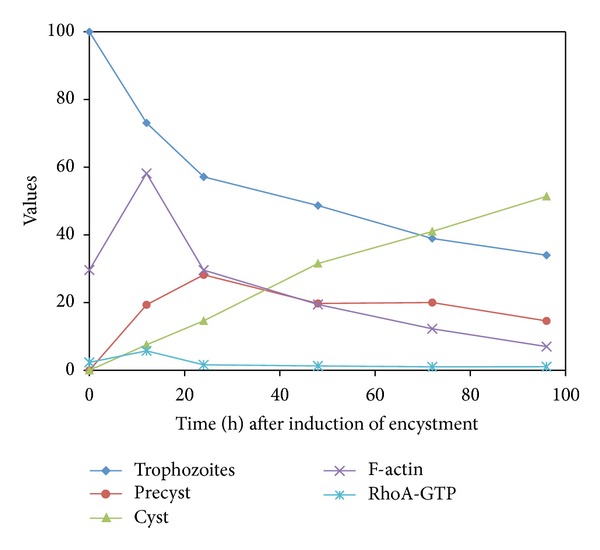
Performance of RhoA-GTP and actin during the transitional stages throughout the encystment kinetic. During the encystment process, trophozoites, precysts, and cysts were quantified and comparatively analyzed with the behavior of F-actin intensity and RhoA-GTP levels (*ρ*g).

Experiments were performed using asynchronous cultures of *E. invadens* due to the difficulty to obtain synchronous cultures, and as a result of this, the encystment process was not uniform. Pleomorphic trophozoites, rounded precysts, and refractive cysts were simultaneously present in most conditions (12–96 h after induction), however, with their respective numbers changing along the time.  Figure 9 shows the percentage of each of these phases during encystment kinetics. The number of cysts increased in relation to time, observing the following percentages: 12 h (7.54 ± 1.1%), 24 h (14.63 ± 5.59%), 48 h (31.56 ± 0.64%), 72 h (41.02 ± 8.61%), and 96 h (53.39 ± 8.01%) after induction of encystment. The higher percentage of precysts was observed at 24 h (28.21 ± 4.19%) (Table 2).

Statistical analysis by linear regression was done, finding that trophozoites decreased proportionally during the time, at a rate of 0.12% per hour (*P* ≤ 0.01), while cysts increased at a rate of 0.54% by each hour (*P* ≤ 0.01). Additionally, we found a correlation between F-actin and RhoA-GTP values; these molecules were related in a direct proportion.

## 4. Discussion

*Entamoeba* genus has an extremely dynamic cytoskeleton, a key to various biological processes as phagocytosis, adhesion, and motility [10]. This cytoskeleton is composed mainly by actin and actin binding proteins (ABPs), and the ratio of polymerized/depolymerized actin is regulated by small GTPases. It is well known that the rearrangement of the actin cytoskeleton is particularly relevant for *E. invadens* encystment [7]; however, the structuration of actin and the functional involvement of RhoA and Rab11 during encystment of *E. invadens* have not been studied yet.

Actin concentration was determined in whole extracts by western blot; no differences could be observed among parasite phases. A constant level of actin in trophozoites, cysts, and metacystic amoebae had also been described in a previous report [20]. However, when subcellular fractions (soluble and insoluble) were analyzed, clear differences could be perceived along the encystment kinetic. These results were confirmed by fluorescence microscopy showing that F-actin significantly increased at 12 h. Actin cytoskeleton was arranged in different structures, suggesting that actin cytoskeleton can participate in various events during the encystment. Transmission electron microscopy data allowed observing that the actin cytoskeleton was surrounding vacuoles. These results suggest that actin cytoskeleton might participate in the transportation and disposal of vacuoles during encystment.

Rho family of GTPases is involved in a variety of biological processes associated with actin cytoskeletal activation. In *E. histolytica*, it has been described that Rho associated protein kinase 2 (ROCK-2) associates with Rho1, corroborating the active state of this protein as well as its participation in actin microfilament rearrangements induced by fibronectin signaling [10]. Moreover, Bosch et al. (2012) proposed that *Eh*Rho1-*Eh*Formin1 signaling axis is essential in the formation of actin rich structures, such as pseudopodia, and also that it participates in phagocytosis and pinocytosis in response to extracellular cues such as serum factors [11]. It has been suggested that in the amoeba *Balamuthia mandrillaris *RhoA participates in the actin polymerization during encystment [28]. In agreement with these reports, the participation of RhoA in the actin cytoskeleton organization during the encystment process is clearly demonstrated in this work, where active state of RhoA increased at early times and decreased at later times after induction. Previous metabolic profiling studies showed, in *E. invadens*, that elevated levels of GTP were present at early times (0.5, 2, and 8 h) and lower levels at later times (24, 48, and 120 h) after induction of encystment with respect to trophozoites [29], indirectly suggesting the presence, among other kinases, of the active state of RhoA at early times after induction of encystment. 

The inhibition of ROCK-2, an effector of Rho, decreased polymerized actin and the number of cysts. Additionally, statistical analysis shows a correlation between F-actin and RhoA-GTP levels. These results suggest that RhoA is activating the rearrangement of the actin cytoskeleton, an important event to carry out the encystment of *E. invadens*. Activation of Rho in fibroblasts leads to stress fiber assembly; however in *E. invadens*, stress fibers were not observed during encystment. It should be noted that the response to Rho GTPase activation varies with cell type; for example, Rho activation induces neurite retraction and neuronal cell rounding without forming stress fibers [30]. Disruption of the actin cytoskeleton rearrangement, by Cytochalasin D and Y27632, was able to prevent some events of encystment such as morphological change, the elimination of vacuoles, the production of encystment vesicles, and the proper formation of the cyst wall. These results confirmed the participation of the actin cytoskeleton in the morphological changes, the transportation and disposal of vacuoles and the cyst wall formation, thus producing a smaller rounded cell protected by a cyst wall.

Enolase (ENO) and Rab11 colocalized in vesicles throughout the process of encystment. However, F-actin, structured as vesicles, was seen only at later times colocalizing with ENO and Rab11. This suggests that components of the cyst wall, such as ENO, could be transported by Rab11 to the plasma membrane. The formation of encystment vesicles (positive for ENO) occurred at the same time with Rab11 relocation from the plasma membrane to these vesicles, and it was until later times that F-actin was observed colocalizing with ENO+/Rab11+ vesicles; this last observation suggests that the transport of vesicles could be through F-actin during cyst differentiation.

## 5. Conclusions

We have demonstrated that the actin cytoskeleton is playing different roles during encystment of *E. invadens*, such as morphological change, transportation and disposal of vacuoles, and transport of encystment vesicles from the cytosol to the cell wall. The actin cytoskeleton reorganization could be activated by RhoA-GTP while Rab11 could be carrying ENO to the cell wall with the actin cytoskeleton help. 

In this work, the functional role of some key molecules that participate during the *E. invadens* differentiation process was described; therefore, these results provide the basis for the development of new strategies to disrupt the cyst differentiation process. However, further studies are required.

## Figures and Tables

**Table 1:** Co-localization coefficient between ENO, Rab11, and F-actin. Co-localization coefficient of pairs of molecules; values are average of the co-localization coefficient of different confocal planes; the highest values from three amoebae were selected.

Time (h)	ENO and Rab11	ENO and F-actin	Rab11 and F-actin
0	0.053 ± 0.025	0.191 ± 0.008	0.088 ± 0.001
12	0.655 ± 0.054	0.036 ± 0.040	0.051 ± 0.015
24	0.780 ± 0.008	0.173 ± 0.070	0.047 ± 0.017
48	0.703 ± 0.035	0.099 ± 0.010	0.119 ± 0.016
72	0.192 ± 0.051	0.127 ± 0.033	0.157 ± 0.037
96	0.194 ± 0.146	0.039 ± 0.017	0.029 ± 0.010

**Table 2:** Average percentage of trophozoites, precysts, and cysts during encystment kinetic, taking the entire population as 100%. Data corresponding to mean percentage ± S.D. are representative of three independent experiments.

Time (h)	Trophozoite	Precyst	Cyst
0	100 ± 0	0 ± 0	0 ± 0
12	73.10 ± 7.40	19.36 ± 7.21	7.54 ± 1.10
24	57.16 ± 5.92	28.21 ± 4.19	14.63 ± 5.59
48	48.68 ± 5.79	19.76 ± 5.64	31.56 ± 0.64
72	38.95 ± 5.29	20.03 ± 3.54	41.02 ± 8.61
96	34.01 ± 6.71	14.60 ± 5.95	51.39 ± 8.01

## Abbreviations

G-actin: Globular actin
F-actin: Filamentous actin
CD: Cytochalasin D
ENO: Enolase
FITC: Fluorescein isothiocyanate
LG: Low glucose
TYI-S: Trypticase-yeast extract-iron-serum
Sf: Soluble fraction
If: Insoluble fraction
RT: Room temperature
DMSO: Dimethyl sulfoxide
HRP: Horseradish peroxidase
ROCK-2: Rho-associated protein kinase 2.
